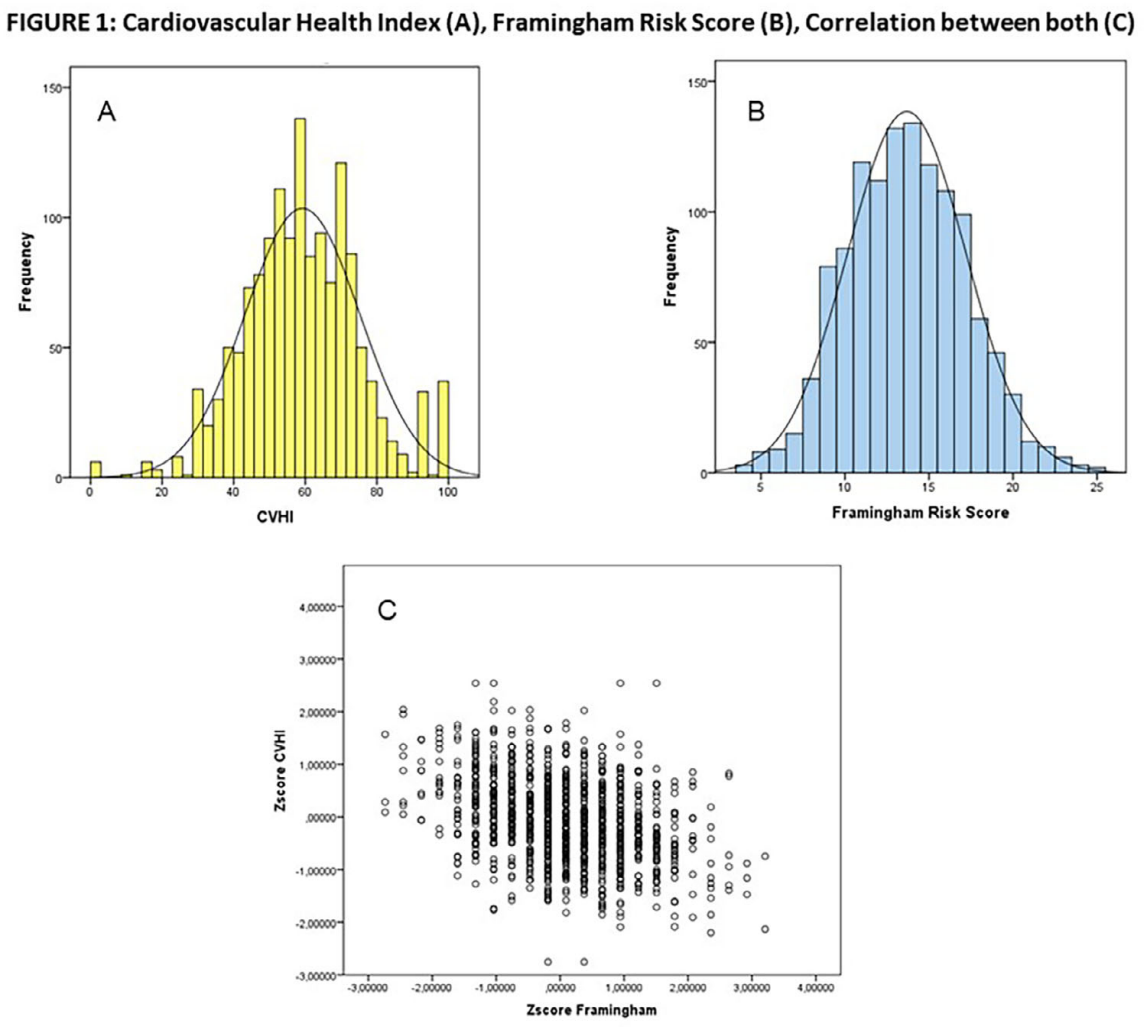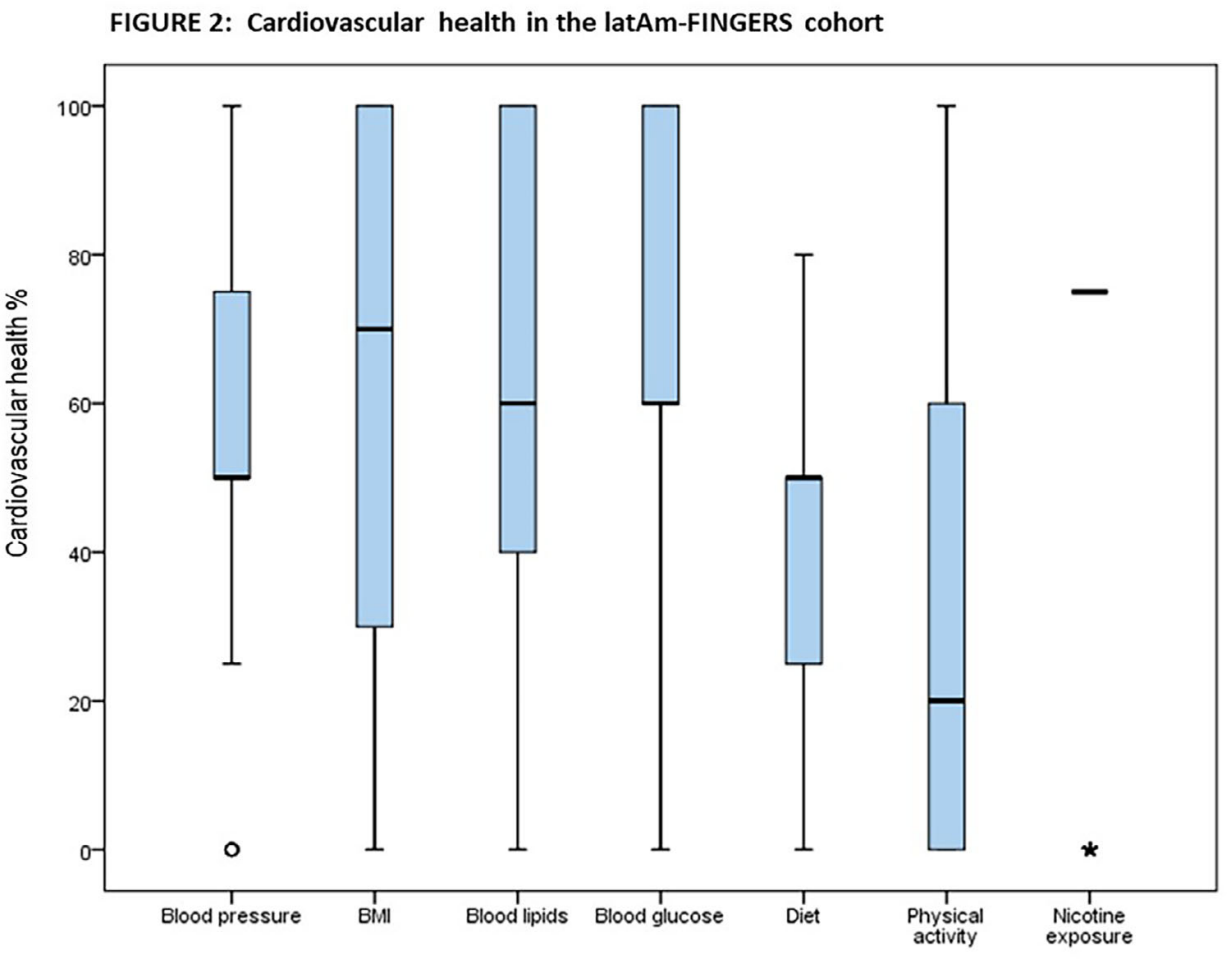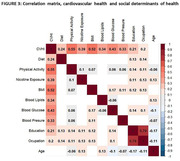# Cardiovascular health is associated with social determinants of health in the LatAm‐FINGERS cohort

**DOI:** 10.1002/alz.092229

**Published:** 2025-01-09

**Authors:** Carolina Delgado, Rodrigo C. Vergara, Natalia Pozo, Lucia Crivelli, Paulo Caramelli, Ricardo Nitrini, Gustavo Sevlever, Ismael Luis Calandri, Carlos Doren, Consuelo San Martin, Javiera Rodriguez, Myriam Gutiérrez, Jacinta Bravo, Maira Moreno, Carlos Márquez, Oriana Lara, Jamileth More, Jose Lema, Rodrigo Gomez, Ricardo Allegri, Francisco Lopera, Lina Marcela Vellila, Monica Yassuda, Ana Luisa Sosa, Rosa Maria Salinas‐Contreras, Claudia Kimie Suemoto, LatAM FINGERS Consortium

**Affiliations:** ^1^ Hospital Clínico Universidad de Chile, Santiago, Region Metropolitana Chile; ^2^ Facultad de Psicología y Humanidades, Universidad San Sebastián, Valdivia Chile; ^3^ Centro Nacional de Inteligencia Artificial CENIA, Santiago Chile; ^4^ Hospital San Borja Arriarán, Santiago Chile; ^5^ Global Brain Health Institute, Memory and Aging Center, University of California San Francisco, San Francisco, CA USA; ^6^ Department of Cognitive Neurology, Montañeses, Buenos Aires Argentina; ^7^ Fleni, Buenos Aires, Buenos Aires Argentina; ^8^ Faculty of Medicine ‐ Universidade Federal de Minas Gerais, Belo Horizonte Brazil; ^9^ Universidade de Sao Pablo, Sao Pablo Brazil; ^10^ Universidad de Chile, Santiago, Metropolitana Chile; ^11^ Universidad de los Andes, santiago, Santiago Chile; ^12^ Hospital Clínico Universidad de Chile, Santiago Chile; ^13^ Instituto de Nutrición y Tecnología de los Alimentos (INTA), Universidad de Chile, Santiago Chile; ^14^ Universidad de Chile, Santiago, metropolitana Chile; ^15^ Geroscience Center for Brain Health and Metabolism (GERO), Santiago, Region Metropolitana Chile; ^16^ Kintun, Santiago, metropolitana Chile; ^17^ Grupo de Neurociencias de Antioquia, Universidad de Antioquia, Medellin Colombia; ^18^ Sao paulo, Sao Paulo Brazil; ^19^ Dementias Laboratory, National Institute of Neurology and Neurosurgery, Mexico City, DF Mexico; ^20^ Laboratorio de demencias del Instituto Nacional de Neurología y Neurocirugía Manuel Velasco Suárez, Ciudad de Mexico Mexico; ^21^ Division of Geriatrics, Department of Internal Medicine, University of Sao Paulo Medical School, São Paulo, São Paulo Brazil; ^22^ University of São Paulo Medical School, São Paulo Brazil

## Abstract

**Background:**

Cardiovascular risk factors (CVRF) are among the main modifiable risk factors for dementia in Latin America (LA). Therefore, improving cardiovascular health (CVH) is one of the main objectives of the LatAm‐FINGERS trial, the largest non‐pharmacological (lifestyle improvement) randomized trial in LA. But, to fully comprehend CVH it is necessary to explore its relation with the social determinants of health (SDH), that are closely associated with lifestyle.

**Methods:**

LatAm‐FINGERS is an initiative to develop a joint regional intervention protocol to prevent cognitive deterioration in 12 LA countries. Participants (between 60‐77 years old) should have high dementia risk (CAIDE >6), were evaluated clinically and cognitively at baseline and every 6 months for 2 years. At baseline, we measured the CVRF according to the Framingham risk score and the CVH with the “Life’s Essential 8 cardiovascular health index” (CVHI), a composed score that includes lifestyle’s (diet, physical activity, nicotine exposure, sleep health) and metabolic variables (body mass index (BMI), blood lipids, blood glucose, and blood pressure). Each score ranges from 0 to 100, with higher values meaning a healthier profile. SDH was characterized by years of education, race, and occupation. Occupation categorical data was transformed into an ordinal scale using the Hollingshead score. Partial correlations (controlled by age and sex) between CVHI and SDH measures were done.

**Results:**

Preliminary data from 1,024 participants were analyzed, age = 67±5 years, education = 13±4 years, 72% women, 87% had high CVRF. The CVHI (60 ± 16) was obtained with 7 of the 8 variables, excluding sleep health (Figure 1). Diet (39±13) and physical activity (37±39) were the unhealthiest scores, while blood glucose (75±26) was the healthiest one (Figure 2). There were significant correlations between CVHI with years of education (r = 0.179, p<0.001) and occupation score (r = 0.169, p<0.001). Moreover, CVH individual components correlated with this SDH, except for blood lipids (Figure 3).

**Conclusions:**

In the LatAm‐FINGERS cohort, better socioeconomic position is associated with a healthier cardiovascular index at baseline, being important to explore the role of SDH in CVH modification across the trial.

Funded by Alzheimer Association